# Integrative Analysis with Monte Carlo Cross-Validation Reveals miRNAs Regulating Pathways Cross-Talk in Aggressive Breast Cancer

**DOI:** 10.1155/2015/831314

**Published:** 2015-07-09

**Authors:** Antonio Colaprico, Claudia Cava, Gloria Bertoli, Gianluca Bontempi, Isabella Castiglioni

**Affiliations:** ^1^Interuniversity Institute of Bioinformatics in Brussels (IB)^2^, 1050 Brussels, Belgium; ^2^Machine Learning Group, ULB, 1050 Brussels, Belgium; ^3^IBFM-CNR, Segrate, 20090 Milan, Italy

## Abstract

In this work an integrated approach was used to identify functional miRNAs regulating gene pathway cross-talk in breast cancer (BC). We first integrated gene expression profiles and biological pathway information to explore the underlying associations between genes differently expressed among normal and BC samples and pathways enriched from these genes. For each pair of pathways, a score was derived from the distribution of gene expression levels by quantifying their pathway cross-talk. Random forest classification allowed the identification of pairs of pathways with high cross-talk. We assessed miRNAs regulating the identified gene pathways by a mutual information analysis. A Fisher test was applied to demonstrate their significance in the regulated pathways. Our results suggest interesting networks of pathways that could be key regulatory of target genes in BC, including stem cell pluripotency, coagulation, and hypoxia pathways and miRNAs that control these networks could be potential biomarkers for diagnostic, prognostic, and therapeutic development in BC. This work shows that standard methods of predicting normal and tumor classes such as differentially expressed miRNAs or transcription factors could lose intrinsic features; instead our approach revealed the responsible molecules of the disease.

## 1. Introduction

In recent years, gene expression experiments identified an increasing number of disease biomarkers, for example, [1–4]. In Breast Cancer (BC) different gene signatures have been identified [5–10], some of them with similar role (e.g., BC grade classification), but their reproducibility and overlap are poor. Gene signatures of many gene-based classification methods are often generated by genes selected independently, even though their functional products may interact with each other, and the selected gene markers may contain redundant information. In this way, they may not synergistically improve the overall classification performance. In order to overcome these limitations, one of the key challenges of the postgenomic era is to understand the complex interaction among genes, thus going a step forward the elucidation of essential principles of cellular systems and disease machinery [11, 12]. In addition, in order to obtain a correct interpretation of high-throughput genomic experiments, the identification of signaling and metabolic pathways involved in a given phenotype is a crucial step. Several studies [13, 14] have shown that pathway-based classifiers are more reproducible and often achieve comparable or better classification performance than classifier based on independent gene biomarkers [15].

Most methods, currently available, consider the pathways as independent mechanisms, and they do not treat the relation between pathways, which is referred to as cross-talk. A cross-talk among gene pathways can be mean of regulatory interaction among different pathways or can express the gene overlap among pathways.

In a normal condition many of the cellular signaling pathways are interconnected to maintain homeostasis [16]. However, the development of cancer might affect the pathway interaction and comprehensively alter the phenotype of a cell. Therefore, the interaction among pathways is a crucial step for understanding the regulatory mechanisms and the synergistic effects on certain biological processes.

Cancer develops through a complex multistep process, involving both genetics and epigenetic mechanisms. Epigenetic alterations include DNA methylation, histone modifications, and small noncoding RNA molecules, including microRNAs (miRNAs). They are involved in the modulation of gene expression on a large scale [17–20]. In recent years, miRNAs have been reported to control many biological processes, such as development, differentiation, growth, and even cancer development and progression [21, 22]. Therefore, to investigate and integrate cross-talk pathways that comprise both genes and miRNAs have become crucial. Recent studies have shown that miRNAs can mediate the cross-talk between pathways. Butz et al. [23] reported cross-talk between TGF-beta signaling and the miRNA machinery to suggest potential novel therapeutic targets. Wang et al. [24] provided an overview description of the cross-talk between Notch signaling with many pathways and evidenced where miRNAs appear to play a major role. Zhao and Carrasco [25] discussed the appealing possibility of a functional link between miRNA30a/b/c/d/e-5p and Wnt/beta-catenin pathway for multiple myeloma therapy.

To our knowledge, there are few studies that examine the role of miRNAs for cross-talked pathway able to correctly discriminate normal versus BC samples. Recently, in myasthenia gravis Cao et al. [26] calculated the cross-talk between pathways, identified by pathway-enriched analysis based on a cumulative hypergeometric distribution. They obtained key genes regulated by miRNAs, and these miRNAs were found to mediate cross-talk.

Many pathways cross-talk was demonstrated in cancer, but few studies were focused on the regulatory role of miRNAs. For instance, Notch signaling pathway showed a cross-talk with multiple oncogenic signaling pathways, such as NF-*κ*B, Akt, Sonic hedgehog (Shh), mTOR, Ras, Wnt, estrogen receptor (ER), androgen receptor (AR), epidermal growth factor receptor (EGFR), and platelet-derived growth factor (PDGF), maybe playing critical roles in tumor aggressiveness [27]. Recently, Hiemer et al. [28] revealed novel cross-talk between the TGF-beta pathway and TAZ/YAP in late-stage BC. Han et al. [29] presented an evidence of cross-talk between TGF-beta signaling and Notch pathway through a basic helix-loop-helix transcription factor, HEYL.

Several studies suggested the potential role of pathway cross-talk in therapeutic approaches [30, 31]. Leehy et al. [30] showed that Aurora A kinase and progesterone receptor (PR) cross-talk may drive early BC progression in response to growth promoting signals. Aurora kinase inhibitors and antiprogestins, administered with conventional estrogen-blocking therapies, may increase survival outcomes by preventing progression to endocrine failure. Recent studies focused on understanding the molecular biology associated with acquired endocrine resistance, like cross-talk between ER and peptide growth factor receptor pathways, such as EGFR and human epidermal growth factor receptor 2 (HER2) [31]. Future therapeutic approaches may combine endocrine therapy with inhibitors of growth factor receptors or downstream signaling pathways to treat or avoid critical resistance pathways that become active in ER+ tumors.

Although it is intuitive that different pathways could influence each other, the presence and amount of this scenario have not been completely studied and, most importantly, there is no currently available technique able to quantify the amount of such cross-talk for pairs of pathway [32].

Donato et al. [32] demonstrated, using Jaccard similarity index and Pearson correlation for pairs of pathways, that the cross-talk among two pathways can be explained by the presence of genes that are involved in more than one pathway. However, they focused on cross-talk due to the effect that pathways exercise on each other for the presence of overlapping genes. They did not explore cross-talk as regulatory interaction among different pathways. Lee et al. [33] proposed a pathway activity score, summarizing the activity level of each pathway from the gene-expression level of its condition-responsive genes (CORGs). CORGs were defined as the subset of genes in the pathway whose combined expression delivers optimal discriminative power for the disease phenotype. Yang et al. [34] proposed an average rank-based score capturing the relative expression levels of genes in a pathway and used Spearman test to evaluate similarity between pathways. Furthermore, they showed that both correlation of activity profiles between pathways and correlations of expression profiles for genes from the same pathways are reduced in tumors with respect to normal samples or cell lines. Guo et al. [35] used the mean or median expression value of the member genes (that belong to the same pathway) as the activity level of a given pathway. Su et al. [15] proposed a probabilistic pathway activity inference method that uses the log-likelihood ratio between different phenotypes based on the expression level of each member gene. Cava et al. [36] presented some distance measures using pathway information among BC patients showing that, although not improving the classification of BC, their possible use in the pathway cross-talk expression could be beneficial for more reproducible and biologically valid gene signatures in BC. McCormack et al. [37] proposed CrossTalkZ, a statistical method to identify the significance of cross-talk enrichment between pairs of gene or protein groups in large biological networks.

**Figure 1:**
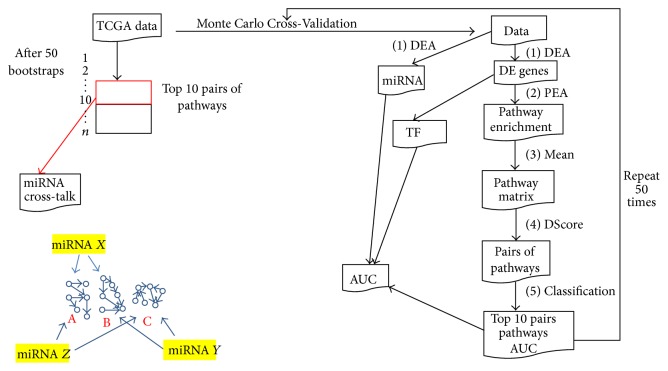
Workflow of the proposed methodology.

In this work our goal was to develop an approach that could be able to quantify the cross-talk between pathways and to identify key miRNAs regulating pathway cross-talk.  Figure 1 shows our proposed methodology. We integrated the information of differentially expressed genes (DEGs) in a Differential Expression Analysis (DEA) (1st step) between BC and normal samples (NS) with the pathway information (2nd step) in a Pathway Enrichment Analysis (PEA). We thus found a subset of pathways enriched from DEGs and, starting from a matrix containing mean of genes for each pathway (3rd step), we created a matrix score for each pair of pathways (4th step). These procedures were tested through a Monte Carlo Cross-Validation (50 bootstraps). A training dataset was used for each bootstrap in order to find a matrix score for the top 10 pairs of pathways with the best AUC value between NS versus BC samples (Random Forest classification). Finally, we considered the top 10 pairs of pathways ranked for all 50 bootstraps (5th step) as those most frequently found. We then focused on miRNAs targeting the higher number of genes within each pathway pairs (6th step). We found an interesting subset of miRNAs that can be involved in the cross-talk regulation among different pathways in aggressive BC. We assessed pairs of pathways that could be key regulatory of BC and miRNAs that control these pairs of pathways as potential biomarkers for the diagnosis of BC.

The paper is organized as follows.  Section 2 covers material and methods by detailing the proposed approach and defining the adopted evaluation procedure. Sections 3 and 4 report and discuss the results obtained by applying the proposed approach on two different datasets.  Section 5 draws our conclusions.

## 2. Materials and Methods

### 2.1. Datasets

We applied our approach on two BC datasets obtained from the Cancer Genome Atlas (TCGA) and from the Gene Expression Omnibus (GEO): GSE39004. TCGA is a comprehensive and coordinated effort to accelerate our understanding of the molecular basis of cancer by providing a huge amount of genomic data obtained with the ultimate genome analysis technologies. The data are organized in four levels: (1) raw, (2) processed, (3) interpreted, and (4) region of interest.

In our approach, we adopted the level 3. We used the package TCGAbiolinks  [38] to create two Expression Matrices, after downloading files of miRNAs and mRNAs from TCGA. The BC dataset contains: (1) the expression levels of 1046 miRNAs from 720 tumor samples and 87 NS, obtained with Illumina Genome Analyzer miRNA Sequencing, which reports the calculated expression for each miRNA sequence isoform observed and (2) the expression level of 15243 genes (after quantile analysis) and 1052 tumor samples and 113 NS obtained with IlluminaHiSeq RNASeqV2, which reports the normalized results for the expression of a gene.

In our analysis, we used 142 BC and 113 NS from BC RNAseqV2, in order to obtain primary BC patients with stage greater than 3 and matched samples between mRNA and miRNA and 142 BC and 87 NS from BC illuminahiseq mirnaseq.

In order to avoid cohort-specific biases, we used also a BC dataset from Gene Expression Omnibus (GEO): GSE 39004. It contains 108 BC samples: 61 samples of macrodissected tumor tissue and 47 adjacent noncancerous tissues. The dataset comes from the Affymetrix Gene Chip Human Gene 1.0 ST Arrays platform.

### 2.2. The Proposed Approach

Our goal was to develop an approach to quantify the cross-talk between pathways and to identify key miRNAs regulating pathway cross-talk.  Figure 1 shows the proposed methodology. In Monte Carlo Cross-Validation approach all steps were repeated for 50 bootstraps. For each bootstrap in our approach we obtained (1) DEA, (2) PEA, (3) Mean, (4) DScore, and (5) Classification. After 50 bootstraps we focused on top 10 pairs of pathways with the best AUC and their miRNAs. Our approach was compared using the genomic biomarkers selected by the Monte Carlo Cross-Validation: transcription factors (TF) and miRNAs from DEA.

*Step 1: Differential Expression Analysis*. We applied the DEA on those mRNA transcripts and miRNA, which had mean across the 142 samples, higher than the 0.25 *∗* quantile mean across all samples. To determine whether a gene or a miRNA is expressed in a differential way, we applied a test of hypothesis and the fold-change between the two starting conditions, in aggressive BC and normal conditions.

In particular, we used the edgeR package from Bioconductor that uses the quantile-adjusted conditional maximum likelihood (qCML) method for experiments with single factor to determine genes differentially expressed [39]. Compared against several other estimators, qCML is the most reliable in terms of bias on a wide range of conditions and specifically performs best in the situation of many small samples with a common dispersion. The *p* values, generated from the analysis sorted in ascending order, are corrected using the Benjamini-Hochberg procedure for multiple testing correction [40]. We considered significant DEGs or miRNA differentially expressed between BC and NS if log fold change (FC) > 1 and FDR < 0.01.

*Step 2: Pathway Enrichment Analysis*. In order to identify a group of pathways significantly enriched by DEGs in BC with respect to NS, we used a Pathway Enrichment Analysis from DEGS (PEA-DEGs). In particular, we focused on 589 biological pathways derived from the IPA (Ingenuity Pathways Analysis) tool (http://www.ingenuity.com/).

The enrichment was evaluated using Fisher's exact test. The aim was to place DEGs within a regulatory context (IPA pathways) and identify the pathways responsible for coordinating their activity, thus highlighting the regulatory apparatus driving phenotypic differentiation. A Fisher's test was applied between DEGs and genes of IPA pathways and we thus obtained pathway enriched with *p* value < 0.01. *p* values were adjusted using the Benjamini-Hochberg procedure for multiple testing correction [40]. IPA pathways were filtered considering only the genes obtained from the quantile function.

*Steps 3-4: Discriminating Score for Pathway Cross-Talk*. We computed a discriminating score (DS) by comparing the gene expression levels of each pair of IPA pathways enriched from DEGs, in each sample (e.g., we applied a DS(*x*, *y*) in each sample for the pair of pathways *x* and *y*). DS was defined as

(1) $$\begin{array}{c} \text{DS}=\frac{\left|\left(M_{x}-M_{y}\right)\right|}{S_{x}+S_{y}}, \end{array}$$

where *M*_*x*_ and *S*_*x*_ represent mean and standard deviation of expression levels of genes in a pathway *x* and *M*_*y*_ and *S*_*y*_ in a pathway *y*. DS score indicates the relationships between pairs of pathway, with a larger value indicating relatively higher difference of activity between pathways. The considered DSs were already used in previous studies by Golub et al. [41] and Orsetti et al. [42] for the comparison of expression levels between the subgroup of samples presenting amplification and the subgroup of samples without amplification. We used the score for the first time at our knowledge for pathway cross-talk analysis.

We compared DS with the method proposed by Cava et al. [36], which uses the Euclidean distance as metric to quantify pathway cross-talk.

*Step 5: Selection of the Best Pairs of Pathways*. In order to evaluate the performance of the proposed methodology, we developed a Random Forest (RF) classification model using the R-package [43]. The model was used to classify the considered BC versus NS. AUC was estimated by cross-validation method (*k*-fold cross-validation, *k* = 10). We adopted the following parameters: mtry (number of variables randomly sampled as candidates at each split) = sqrt(*p*), *p* being the number of variables in the matrix of data; ntree (number of trees grown) = 500. Classification was applied on pairs of pathways using DS for each sample.

We implemented a Monte Carlo Cross-Validation method. It randomly selected some fraction of TCGA data (60% of original dataset) to form the training set and then assigned the rest of the points to the testing set (40% of original dataset). This process was then repeated multiple times (50 bootstraps), generating (at random) new training and test partitions each time. For each bootstrap we analyzed DEGs, pathways PEA-DEGs, and a matrix score for pairs of pathways.  Figure 1 summarizes the procedure. Each bootstrap gives pairs of pathway significantly enriched from DEGs and from a matrix score (DS); RF classifier establishes an AUC value. We thus considered the top 10 pairs of pathway for each bootstrap that obtained the best classification performance in the training dataset. For each bootstrap a testing dataset was then used to validate the top 10 pairs of pathways. At the end of all 50 bootstraps (runs) we selected a list of the top 10 pairs of pathways ordering according a decreasing frequency that each pair of pathway was selected in the 50 runs.

We ordered each pathway with respect to their AUC and we got the first 10 pathways with the best 10 AUC. Specifically, we considered for further analysis top 10 pairs with better ranked AUC value for all 50 bootstraps.  Figure 1 shows how the selection of the top 10 pairs of pathways is performed for each bootstrap and the final result after 50 bootstraps in the training dataset.

*Step 6: miRNA Regulating Pathway Cross-Talk*. Consider the following.

*(1) miRNA Regulon Estimation*. Network inference, which is the reconstruction of biological networks from high-throughput data, can provide valuable information about the regulation of gene expression in cells. Several methods have been proposed in literature [44], such as TIGRESS and Lasso based on linear regression, Aracne and CLR based on mutual information, correlation, and Bayesian networks. We adopted mutual information as it is particularly effective for large datasets [20, 45, 46]. The mutual information provides an index of dependence between miRNAs and genes.

For each gene belonging to the top 10 pairs of pathways obtained for all 50 bootstraps, we analyzed their miRNAs. We calculated mutual information between dataset of miRNAs and genes, thus creating an index of dependence between them. Mutual information was calculated using entropy estimates from *K*-nearest neighbor distances [47] with the R-package: Parmigene [48]. Mutual information was applied in previous studies [20, 45, 46].

*(2) miRNA Master Regulator Analysis*. Master Regulator Analysis (MRA) [49] is an algorithm used to identify transcription factors whose targets (e.g., as represented in an ARACNe-generated interactome) are enriched for a particular gene signature (e.g., a list of differentially expressed genes). The enrichment is evaluated using a statistical test such as Fisher exact test or Gene Set Enrichment Analysis (GSEA) [50]. The objective is to place the signature genes within a regulatory context and to identify the master regulators responsible for coordinating their activity, thus highlighting the regulatory apparatus driving phenotypic differentiation. We identified miRNA master regulators (MRs) of pair of pathways. A miRNA was defined as MR, when its targets are enriched for a particular gene signature, such as genes involved in one of the two coupled pathways at the same time. Namely, we modified MRA instead of TFs, we used miRNAs, and instead of DEG we used genes annotated in a pathway. We used a Fisher's exact test to identify miRNAs significantly enriched by their target genes from the top 10 pathways as obtained for all 50 bootstraps. We found miRNAs that have a significant target genes in the pair of pathways with both *p* values < 0.01. *p* values were adjusted using the Benjamini-Hochberg procedure for multiple testing correction [40]. Thus, we then focused on miRNAs found differentially expressed in Section 2.2.

*Step 7: Comparison with Other Approaches*. The input of the classifier for our approach was applied on

- DS obtained from each couple of pathways for each sample.

Our approach was compared using the genomic biomarkers selected by the Monte Carlo Cross-Validation. The inputs of the classifier for the comparison were

- the expression levels of TFs in DEGs as obtained from DEA in Section 2.2.
- The resource of transcription factors in human is obtained from TRANSFAC database [51],
- the expression levels of miRNAs as obtained from DEA in Section 2.2.

## 3. Results

### 3.1. Steps 1-2

From quantile analysis, we obtained 15243 genes and 764 miRNAs. From the DEA between aggressive BC and NS for all samples BC TCGA we obtained 3225 DEGs and 254 differentially expressed miRNAs, and a list of 48 significantly pathways enriched from DEGs in total represented a list of 2214 unique genes.  Table 1 shows pathways significantly enriched by DEGs with their FDR score, number of genes for each pathway, and number of common genes between DEGs and genes in the pathway.

### 3.2. Steps 3-4

**Figure 2:**
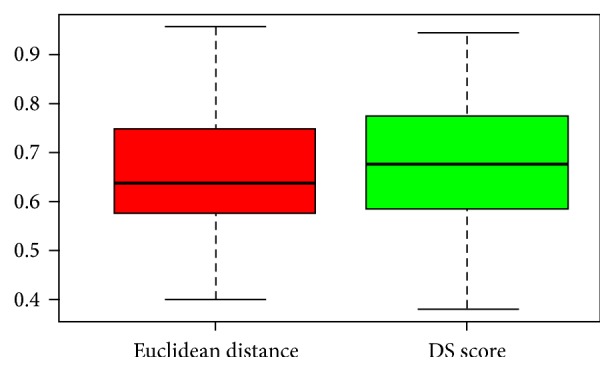
Boxplot of AUC with two different approaches: red (euclidean distance) and DS (green) in the TCGA dataset.

Figure 2 shows AUC classification in the two different approaches. DS obtained a better performance than Euclidean distance. Median values are around 0.65 with euclidean distance and 0.7 with DS.

### 3.3. Step 5

**Figure 3:**
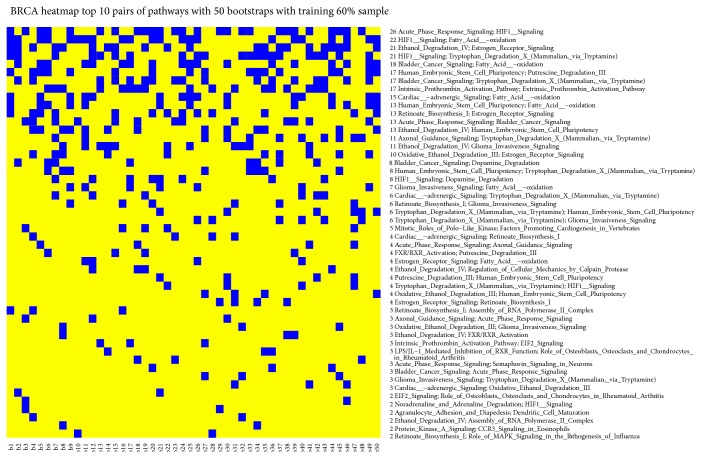
Heatmap with top 10 pairs of pathways (blue square) that obtained the best AUC values in the training dataset.

Figure 3 shows the results for each bootstrap. We obtained a heatmap where blue square indicates pairs of pathways in the top 10 positions for classification in the training dataset for that bootstrap. We found (1) the pair of pathways Acute Phase Response Signaling and HIF1 Signaling in 26 bootstraps; (2) the pair of pathways HIF1 Signaling and Fatty Acid oxidation in 22 bootstraps; (3) the Ethanol Degradation IV and Estrogen Receptor Signaling in 21 bootstraps; and (4) the HIF1 Signaling and Tryptophan Degradation X (Mammalian, via Tryptamine) in 21 bootstraps.

**Figure 4:**
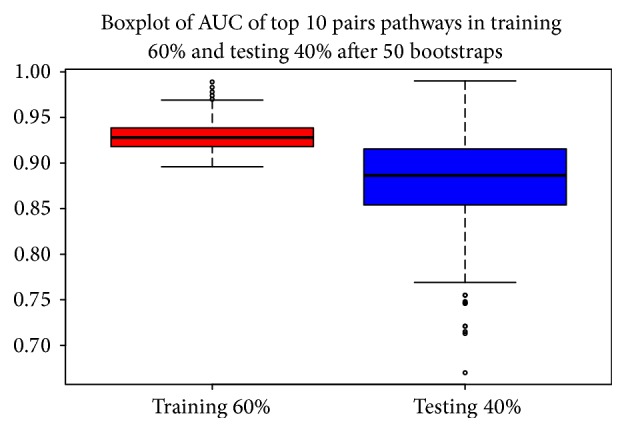
Boxplot: overall AUC performance on both TCGA training (red) and testing dataset (blue).

We ordered each pathway with respect to their AUC and we found 32 paired pathways with AUROC > 0.90 between BC and NS. We focused on pathways with the best 10 AUC.  Table 2 presents the top 10 pairs of pathways that have the best performance for classification of aggressive BC and NS for all 50 bootstraps. It showed AUC value for TCGA and GSE39004 dataset. The pair of pathways, Acute Phase Response Signaling and HIF1 Signaling, with its DScore that was in the top 10 in 26 bootstraps, obtained an AUC value 0.92 in TCGA dataset and 0.889 in GSE39004 dataset, showing a good balance of performance between the two datasets. The pair of pathways Bladder Cancer Signaling and Fatty Acid oxidation showed also a good performance with 0.932 in TCGA dataset and 0.84 in GSE39004 dataset; similar performance was found in the pair of pathways Bladder Cancer Signaling and Tryptophan Degradation X (Mammalian, via Tryptamine) with 0.91 in TCGA dataset and 0.802 in GSE39004 dataset. In TCGA dataset we obtained a mean AUC value 0.92 while in GSE39004 0.70.  Figure 4 shows overall AUC performances when we give only top 10 pairs of pathways for each bootstrap after 50 bootstraps. Results for AUC values are good; 25th percentile does not fall down 0.95.

### 3.4. Step 6: miRNA Regulating Pathway Cross-Talk

We identified 12 miRNAs that may have an important role in the regulation of the four pairs of pathways.

Table 3 reports for each pair of pathways their potential miRNAs together with the log fold change (FC) obtained from the DEA, the miRNA expression levels in BC and NS, and the delta value, an index to quantify the difference of expression between BC and NS for each miRNA (e.g., (ex.BC − ex.NS)*∗*log⁡FC; difference of expression between BC and NS multiplied log⁡FC).* hsa-let-7c*,* hsa-mir-210*, and* hsa-mir-9-1*, according to the Delta index, showed a higher difference between NS and BC.

Table 4 reports for each pair of pathways their potential regulatory miRNA, the number of genes for each pathway, and the number of the potential target genes.  Table 5 reports for pathways their potential regulatory miRNAs and the more frequent genes target of their miRNAs.

**Figure 5:**
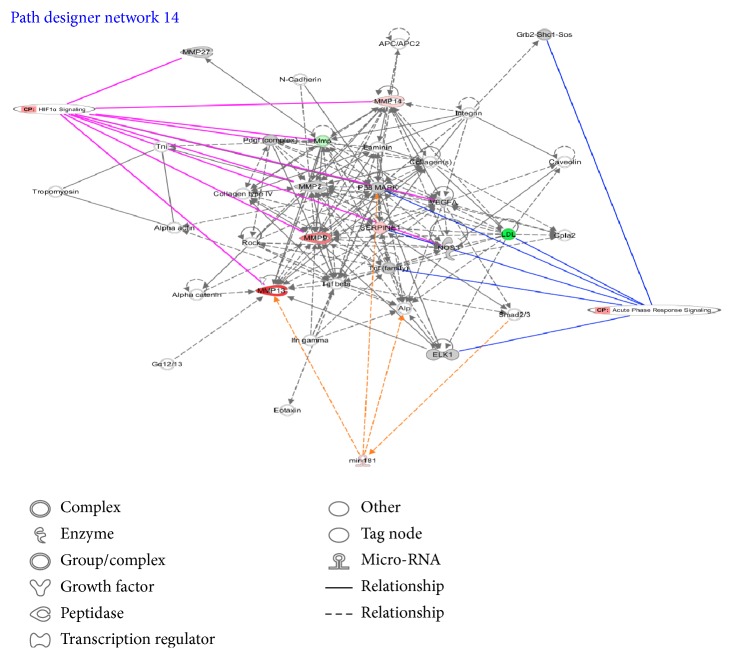
Examples of IPA software outcome, the pair of pathways Acute Phase Response Signaling and HIF1 Signaling, are regulated by* hsa-mir-181*.

**Figure 6:**
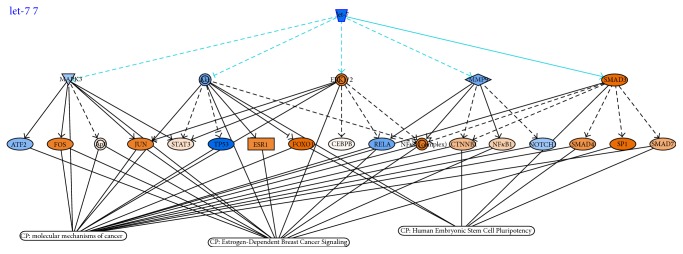
Examples of IPA software outcome: hsa-let-7 and their target genes in Human Embryonic Stem Cell Pluripotency Pathway.

*hsa-mir-181b-2* seemed to regulate the pair of pathways that obtained the best AUC values in both TCGA dataset and independent GSE39004 dataset.  Figure 5 shows the network context of the two pathways Acute Phase Response Signaling and HIF1 Signaling and the possible role of* hsa-mir-181b-2* using IPA software.  Figure 6 shows* hsa-let-7c* and target genes in Human Embryonic Stem Cell Pluripotency, Estrogen-Dependent Breast Cancer Signaling, and Molecular Mechanism of Cancer, according to IPA software.

### 3.5. Step 7: Comparison with Other Approaches

**Figure 7:**
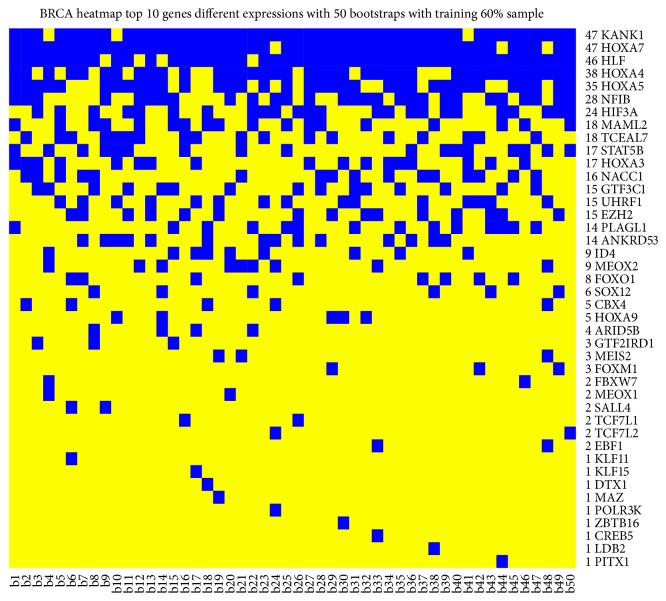
Heatmap with top 10 genes (blue square) that obtained the best AUC values in the training dataset.

**Figure 8:**
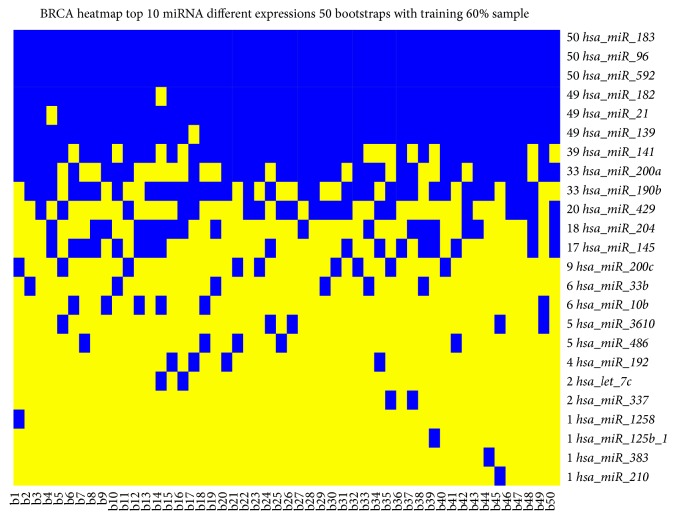
Heatmap with top 10 miRNAs (blue square) that obtained the best AUC values in the training dataset.

**Figure 9:**
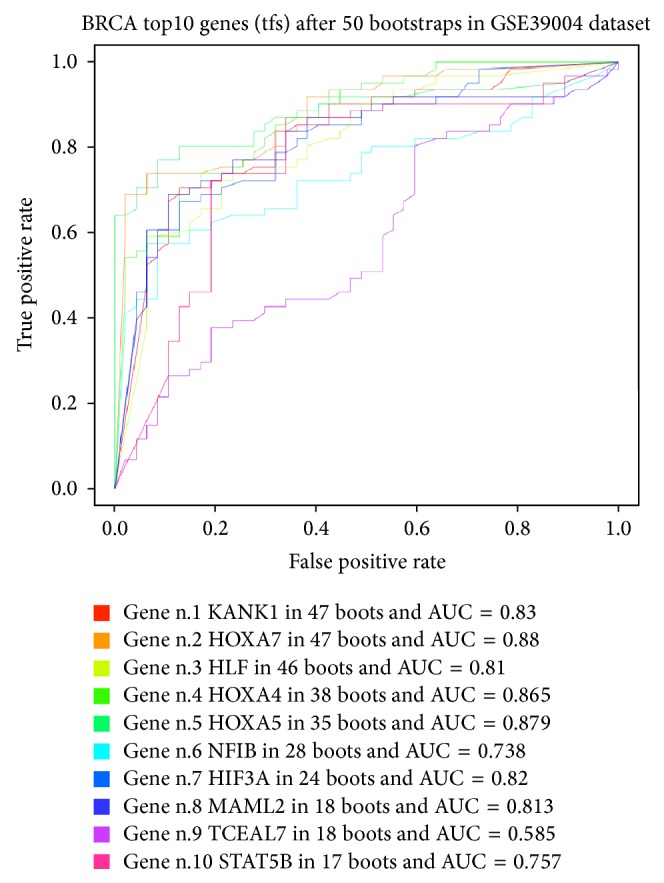
AUC curve for each top 10 genes for all 50 bootstraps.

**Figure 10:**
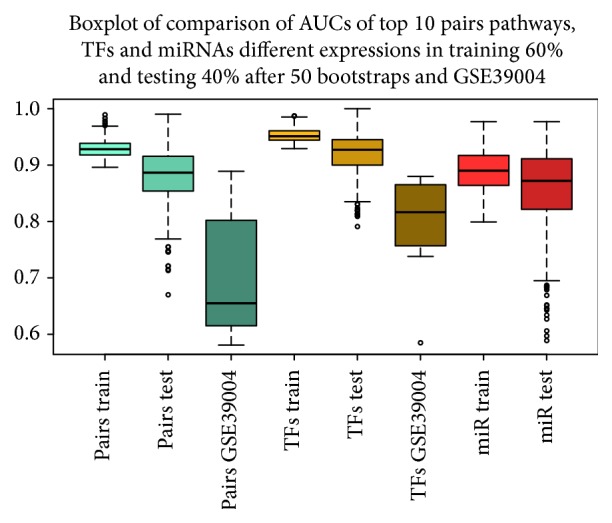
Comparison Boxplot among our approach (pairs train, pairs test, and GSE39004), TF (TFs train, TFs test, GSE39004), and miRNA (miRNA train and miRNA test).

Figures 7 and 8 show DEGs and differentially expressed miRNAs as obtained from Monte Carlo Cross-Validation for each bootstrap. We obtained a heatmap, where blue square indicates DEGs (Figure 7) and miRNAs (Figure 8) in the top 10 positions for classification for that bootstrap.  Figure 9 shows AUC value for top 10 genes that obtained the best classification for all 50 bootstraps in GSE39004.  Figure 10 shows a boxplot with AUC value comparison for each approach. All methods have a good performance. GSE39004 has a poorer AUC, consistently with the limited number of samples available for the *k*-fold validation.

## 4. Discussion

Given the substantial difference in the activities of many pairs of pathways between BC and NS, we examined the effectiveness to classify BC and NS based on their pairwise activity profiles. The final purpose of our work was to find, for the best pairs of pathways able to discriminate BC versuss NS, their miRNA regulators. Different pathways often act in a coordinated manner to participate in many biological process. We calculated DScore to understand the interrelationships among pathways PEA-DEGs between BC and NS.

We applied a RF classifier, using the DS obtained by DEGs of paired pathways significantly associated, and through Monte Carlo Cross-Validation we identified the top 10 pairs of pathways that obtained the best classification for all the considered bootstraps (50).

We found 32 paired pathways with AUROC > 0.90 between BC and NS, but we focused on pathways with the best 10 AUC. From these pairs, by a mutual information approach, we found 4 pairs of pathways potentially targeted by 12 miRNAs.

DS obtained a slightly more improvement than the Euclidean distance, as measure to quantify the cross-talk. Although the performances of our approach with pairs of pathways are similar to these of standard investigations (DEGs and miRNAs) based on traditional gene and miRNA expression analysis, some novel aspects are needed to be investigated:

1. Our method to select the epigenetic signatures (based on the pairs of pathway combinations) has been implemented for the first time, to our knowledge in BC diagnosis with a robust Monte Carlo Cross-Validation approach.
2. Our proposed methodology could be very useful for understanding the interactions between miRNAs and pathways cross-talk. Further studies should be conducted to these purposes.
3. Standard approach with miRNA expression analysis using the same dataset does not found the same our miRNAs give a missing information. For instance,* hsa-let-7c* and* hsa-mir-210* that in our approach could be important miRNA regulating pathway cross-talk in the miRNA analysis are found as differentially expressed only in 2 and 1 bootstraps with the best AUC, respectively (Figure 8).
4. A reduced number of miRNAs could be suitable to be translated in a clinical environment, acting on important network of pathways.
5. This kind of approach is even more interesting from a biological point of view, as the study of the pairs of pathways involved in BC could help in defining the molecular mechanisms leading to the onset and progression of the pathology, a feature that a single TF or a miRNA does not allow understanding.

### 4.1. miRNAs Regulating Pathway Cross-Talk in BC with Stage Greater Than 3

*hsa-mir-181b-2: Acute Phase Response Signaling and HIF1 Signaling*. The pair of pathways Acute Phase Response Signaling and HIF1 Signaling that obtained a good performance in both TCGA dataset and GSE39004 (AUC value 0.92 and 0.88, resp.) seems to be regulated by* hsa-mir-181b-2*. Acute Phase Response Signaling pathway contains 146 genes, hypoxia-inducible factor 1 (HIF-1) signaling contains 94 genes and 25 common genes among them.

We found that* hsa-miR-181b* upregulation is able to control the expression of 26 over 146 genes belonging to Acute Phase Response Signaling and 19 over 94 genes belonging to HIF1 pathway. Altered expression of* hsa-miR-181b* has been found in various malignancies, especially associated with poorer clinical prognosis [52–54]. Several publications linked the inflammatory process with hypoxia pathway [55, 56], but none has demonstrated the link between the two pathways and* hsa-miR-181* expression (Figure 5). In our analysis hsamiR-181 has been proposed as a novel marker for inflammatory response, as its upregulation is strongly correlated with the expression of interleukin (IL)-1*β*, IL-6, and tumor necrosis factor alpha [57]. Recently, it seems that the production of IL-1b induces the expression and secretion of the stem cell factor, a growth factor involved in the control of the proliferation of epithelial BC cells [58]. It is thus possible that the* hsa-miR-181* expression, modulating IL-1b levels, could influence the proliferation of BC, by increasing the production of the stem cell factor.

The acute phase response is a rapid inflammatory response that provides protection against infections [59]. The presence of this pathway in our analysis complies with the generally accepted observation that inflammation is often observed in tumors and appears to play a dominant function in the pathogenesis of various cancer types [59]. Oxygen homeostasis underlies many developmental and physiological processes. Important consequences of rapid tumor growth include poor vascularization and insufficient oxygen delivery involving formation of hypoxic (poorly oxygenated) areas [60, 61]. Adaptation to hypoxia is facilitated by the activation of transcriptional machinery, in which hypoxia inducible factor (HIF) plays a principal role in coordinating angiogenesis. Several studies found cross-talk between HIF-1 signaling and inflammatory pathways suggesting that the development of inflammation in response to hypoxia is clinically relevant [62]: HIF-1 plays a crucial role in hypoxic T-cell and neutrophil survival, an important determinant of tissue inflammation [63]; Zampell et al. [64] found that HIF-1*α* inhibition by small molecule inhibitors (YC-1 and 2-methyoxyestradiol) results in delayed lymphatic repair, decreased local vascular endothelial growth factor-C (VEGF-C) expression, reduced numbers of VEGF-C+ cells, and reductions in inflammatory lymphangiogenesis. Cramer et al. [65] have shown that HIF-1 overexpression is essential for myeloid cell-mediated inflammation. A recent review [62] discussed the regulation of immune responses by hypoxia-induced signaling and the emergent molecular aspects between hypoxia and inflammation in certain cancers.

As shown in Table 5, among genes regulated by* hsa-miR-181*, in both pathways members of the RAS family are present. RAS family members are already described to be involved in BC development [66]. It is relevant that HIF1 activity could influence the activation of RAS/MAPK/ERK1 [67].

*hsa-mir-103-2: Ethanol Degradation IV and Estrogen Receptor Signaling*.* hsa-mir-103-2* corresponds to* hsa-mir-103/107* human homologous and has been already found upregulated in human BC cells, associated with metastatic process [68], and in serum of BC patients by next-generation-sequencing technique [69]. In BC,* hsa-mir-103/107* has an important role of epithelial mesenchymal transition (EMT) regulator [70]. EMT is a key process for metastatic spreading of the BC cells. Our analysis reveals that this miRNA is able to modulate the expression of 4 over 17 genes involved in ethanol degradation and 15 over 112 involved in ER signaling pathway. Looking to the list of target genes of* hsa-mir-103*, we found that a lot of these genes belong to the family of ALDH genes, and, although having a role in ethanol detoxification, are also considered biomarkers of cancer stem cells (CSC) [71]. It is not thus surprising that these genes and their upstream miRNA,* hsa-mir-103*, are associated with higher grade BC samples. In fact, this tumor grade BC contains probably a smaller population of highly invasive and aggressive CSCs [72].

This hypothesis is further supported by the fact that among the top ten cross-talk pathways depicted in Table 2, Human Embryonic Stem Cell Pluripotency Pathway is among the more discriminating metabolic processes between BC and NS.* hsa-mir-103* expression has been already associated with ER status of BC samples, being more abundant in ER+ BC [73].

As shown in Table 5, among the genes controlled by* hsa-mir-103* in estrogen receptor signaling pathway, the family of MED complex is emerging (i.e., MED6, MED15, and MED16). This family contains several members of coactivators of transcription of RNA polymerase II-controlled genes. These proteins have a known regulatory role in metastatic process, as demonstrated by the silencing of MED15 that decreases the metastatic potential of a highly aggressive BC cell line by reducing TGFB/Smad signaling [74].

*Intrinsic Prothrombin Activation Pathway and Extrinsic Prothrombin Activation Pathway*. We found that two cross-talked pathways, extrinsic and intrinsic prothrombine pathways, are regulated by a group of 9 miRNAs (*hsa-miR-147b, hsa-miR-210, hsa-miR-301b, hsa-miR-483, hsa-miR-592, hsa-miR-665, hsa-miR-887, hsa-miR-9-1, *and* hsa-miR-939*). The frequency of miRNA regulation over the genes belonging to the two pathways goes from 33% (3/13) up to 66% (8/12). Analyzing the functional role of the pathways, in almost every patient with a progressive metastasized tumor, a constitutive activation of the coagulation cascade can be found [75]. The coagulation pathway is essential for the establishment of metastasis also in experimental model of cancer. Tumor cells express factors that trigger coagulation in different ways [76]. The extrinsic pathway is triggered by tissue factor (TSF) expressed on the surface of the tumor cells, on microparticles released by the tumor cells, or on the tumor stroma, leading to fibrin formation. TSF binds and activates factor VIIa, initiating the coagulation cascade and leading to thrombin activation. Some of the TSFs involved in the control of the intrinsic pathway are able also to activate the extrinsic pathway. For example, the FXIIa, a factor involved in the intrinsic pathway, indirectly facilitates the extrinsic pathway by converting FVII to FVIIa. The activation of FVII also occurs through the action of thrombin or FXa, mostly generated though the intrinsic pathway. The ability of FXa to activate FVII creates a link between the intrinsic and extrinsic pathways. In reality, the main function of the extrinsic pathway is to magnify the activity of the intrinsic pathway. Metastatic cancer cells have been found to express exceptionally high levels of TSF (up to 100-fold higher than nonmetastatic cells) [77] and it has been suggested that cancer stem cells (CSCs) may express higher levels of TSF [78, 79]. Enhanced cancer cell TSF expression can also lead to increased tumor growth. This may be due to signaling via proteases from tumor environment, such as FVIIa, FXa, or thrombin [80] or to signaling through the TSF cytoplasmic domain [81], which contribute to prooncogenic signals affecting mainly the CSC behavior [82, 83]. The expression of these procoagulant molecules guides development of inappropriate coagulation in cancer, leading to the onset of coagulopathies related with BC [84]. Several are the miRNAs able to control genes belonging to both intrinsic and extrinsic coagulation pathways. Among them,* hsa-miR-210* emerges as one of the miRNAs, whose expression is more altered in higher grade BC [85]. Also the altered expression of other miRNAs, such as* hsa-miR-301b* or* hsa-miR-9-1*, has been found in our miRNA analysis and has been already reported in BC, being associated with proliferation and invasion control [86] or stem cell phenotype [87], respectively.

*hsa-let-7c: Human Embryonic Stem Cell Pluripotency and Putrescine DegradationIII*. The last couple of pathways able to discriminate among BC and NS is the couple formed by Human Embryonic Stem Cell Pluripotency genes and those involved in the process of putrescine degradation. Putrescine is a known metabolite that plays an important role in cancer and cancer stem cells [88]. Putrescine belongs to the class of polyamine that has been shown to affect numerous processes in normal and cancer cells, such as proliferation, apoptosis, cell-cell interactions, and angiogenesis [89]. Total polyamine levels are higher in highly proliferative cells, like cancer cells, and lower in cells with low proliferation rates [89]. It is not surprisingly to find that putrescine degradation pathway is one of the pathways important to distinguish BC from NS.* hsa-let-7c*, the miRNA regulating this metabolite, has a known role in cancer stem cell phenotype control. In particular, it has been reported that altered levels of* hsa-let-7c* are associated with higher grade BC [9, 90], as* hsa-let-7c* is responsible for tumor proliferation control [91]. Emerging evidences have linked the cellular levels of polyamines, such as putrescine, to the regulation of the level of expression of let-7 family members. In particular, polyamines are emerging as potential oncometabolites that influence specific aspects of tumorigenesis by regulating pluripotency associated factors, such as LIN28 [92]. Our speculation is that, in more aggressive BC samples, an alteration in polyamine metabolism alters the level of expression of* hsa-let-7c*, which in turn regulates the pluripotency capacity of the cancer stem cell population. In this way we can explain the link among the two BC-discriminating pathways, the putrescine degradation pathway and the stem cell pluripotency pathway, and the altered level of expression of* hsa-let-7c*.

As shown in Table 5, among the genes regulated by* hsa-let-7c* in Human Embryonic Stem Cell Pluripotency, some are already known stem cell transcription factors, necessary to induce or maintain pluripotency potential (i.e., WNT11 or WNT6, BMP5, and BMP6) [93, 94]. Among the genes controlled by* hsa-let-7c* in putrescine degradation pathway, several of them are related to stem cell phenotype (i.e., the family of aldehyde dehydrogenase, such as ALDH1A1, ALDH1A21, ALDH2, ALDH4A1, ALDH9A1) [95].

## 5. Conclusions

In conclusion, the approach used in our work allowed identifying (1) four pairs of pathways able to accurately classify aggressive BC versus NS with an important role in the regulations of several mechanisms in BC; (2) an epigenetic signature of 12 miRNAs able to regulate those pairs of pathways in BC, and (3) a discriminating score able to quantify the cross-talk among pathways, with a potential diagnostic and therapeutic roles in BC. These interesting suggestions, obtained by in silico analysis, should be further validated in laboratories by using, for example, BC cell lines. Due to the reduced number of miRNAs, our epigenetic signature could be suitable to be translated in a clinical environment.

## Figures and Tables

**Table 1:** Pathways enriched from differentially expressed genes.

Pathway	FDR	Number of genes in pathway	Number of common genes
Agranulocyte Adhesion and Diapedesis	FDR = 6.38*e* − 10	(ng = 173)	(nc = 62)
Atherosclerosis Signaling	FDR = 6.25*e* − 08	(ng = 119)	(nc = 44)
Granulocyte Adhesion and Diapedesis	FDR = 4.26*e* − 07	(ng = 163)	(nc = 53)
LXR/RXR Activation	FDR = 6.58*e* − 07	(ng = 121)	(nc = 42)
Inhibition of Matrix Metalloproteases	FDR = 9.55*e* − 06	(ng = 38)	(nc = 19)
Hepatic Fibrosis/Hepatic Stellate Cell Activation	FDR = 1.20*e* − 05	(ng = 137)	(nc = 44)
Acute Phase Response Signaling	FDR = 2.38*e* − 05	(ng = 167)	(nc = 49)
ILK Signaling	FDR = 4.95*e* − 05	(ng = 181)	(nc = 52)
Ethanol Degradation II	FDR = 7.41*e* − 05	(ng = 30)	(nc = 15)
Intrinsic Prothrombin Activation Pathway	FDR = 1.26*e* − 04	(ng = 28)	(nc = 14)
Coagulation System	FDR = 1.26*e* − 04	(ng = 35)	(nc = 16)
Extrinsic Prothrombin Activation Pathway	FDR = 1.26*e* − 04	(ng = 16)	(nc = 10)
Noradrenaline and Adrenaline Degradation	FDR = 1.36*e* − 04	(ng = 32)	(nc = 15)
Bladder Cancer Signaling	FDR = 1.36*e* − 04	(ng = 86)	(nc = 29)
Axonal Guidance Signaling	FDR = 1.44*e* − 04	(ng = 421)	(nc = 98)
Ethanol Degradation IV	FDR = 1.96*e* − 04	(ng = 17)	(nc = 10)
Colorectal Cancer Metastasis Signaling	FDR = 2.46*e* − 04	(ng = 232)	(nc = 60)
Wnt/_-catenin Signaling	FDR = 2.89*e* − 04	(ng = 167)	(nc = 46)
Cardiac_-adrenergic Signaling	FDR = 4.24*e* − 04	(ng = 132)	(nc = 38)
Protein Kinase A Signaling	FDR = 6.29*e* − 04	(ng = 365)	(nc = 84)
LPS/IL-1 Mediated Inhibition of RXR Function	FDR = 7.24*e* − 04	(ng = 210)	(nc = 52)
EIF2 Signaling	FDR = 1.20*e* − 03	(ng = 171)	(nc = 11)
Epithelial Adherens Junction Signaling	FDR = 1.47*e* − 03	(ng = 144)	(nc = 38)
HIF1_Signaling	FDR = 1.67*e* − 03	(ng = 100)	(nc = 29)
Oxidative Ethanol Degradation III	FDR = 1.67*e* − 03	(ng = 15)	(nc = 8)
Retinoate Biosynthesis I	FDR = 1.67*e* − 03	(ng = 29)	(nc = 12)
Factors Promoting Cardiogenesis in Vertebrates	FDR = 1.74*e* − 03	(ng = 87)	(nc = 26)
GADD45 Signaling	FDR = 2.15*e* − 03	(ng = 19)	(nc = 9)
Mitotic Roles of Polo-Like Kinase	FDR = 2.15*e* − 03	(ng = 62)	(nc = 20)
TR/RXR Activation	FDR = 2.17*e* − 03	(ng = 85)	(nc = 25)
Aryl Hydrocarbon Receptor Signaling	FDR = 2.61*e* − 03	(ng = 133)	(nc = 35)
Role of Osteoblasts, Osteoclasts …	FDR = 3.18*e* − 03	(ng = 214)	(nc = 50)
Estrogen-mediated S-phase Entry	FDR = 3.30*e* − 03	(ng = 24)	(nc = 10)
Calcium Signaling	FDR = 3.64*e* − 03	(ng = 168)	(nc = 41)
Estrogen Receptor Signaling	FDR = 3.73*e* − 03	(ng = 126)	(nc = 8)
Triacylglycerol Biosynthesis	FDR = 4.27*e* − 03	(ng = 33)	(nc = 12)
Mitochondrial Dysfunction	FDR = 4.27*e* − 03	(ng = 139)	(nc = 10)
Thyroid Cancer Signaling	FDR = 4.27*e* − 03	(ng = 40)	(nc = 14)
Human Embryonic Stem Cell Pluripotency	FDR = 4.61*e* − 03	(ng = 132)	(nc = 34)
FXR/RXR Activation	FDR = 5.24*e* − 03	(ng = 82)	(nc = 22)
Glycogen Degradation II	FDR = 8.40*e* − 03	(ng = 9)	(nc = 5)
Putrescine Degradation III	FDR = 8.49*e* − 03	(ng = 16)	(nc = 7)
Tryptophan Degradation X	FDR = 8.49*e* − 03	(ng = 16)	(nc = 7)
Dopamine Degradation	FDR = 9.05*e* − 03	(ng = 21)	(nc = 8)
Glioma Invasiveness Signaling	FDR = 9.89*e* − 03	(ng = 57)	(nc = 17)
Actin Cytoskeleton Signaling	FDR = 9.89*e* − 03	(ng = 210)	(nc = 47)
NAD biosynthesis II (from tryptophan)	FDR = 9.89*e* − 03	(ng = 13)	(nc = 6)
Fatty Acid_-oxidation	FDR = 9.89*e* − 03	(ng = 13)	(nc = 6)

**Table 2:** Top 10 pairs of pathways with AUC value for TCGA testing and GEO dataset.

Pairs of pathways	TCG AUC	GSE39004 AUC
(1a) Acute Phase Response Signaling		
(1b) HIF1 Signaling	0.92	0.889
(2a) HIF1 Signaling		
(2b) Fatty Acid oxidation	0.955	0.654
(3a) Ethanol Degradation IV		
(3b) Estrogen Receptor Signaling	0.925	0.656
(4a) HIF1 Signaling		
(4b) Tryptophan Degradation X (Mammalian, via Tryptamine)	0.922	0.629
(5a) Bladder Cancer Signaling		
(5b) Fatty Acid oxidation	0.932	0.84
(6a) Human Embryonic Stem Cell Pluripotency		
(6b) Putrescine Degradation III	0.931	0.613
(7a) Bladder Cancer Signaling		
(7b) Tryptophan Degradation X (Mammalian, via Tryptamine)	0.91	0.802
(8a) Intrinsic Prothrombin Activation Pathway		
(8b) Extrinsic Prothrombin Activation Pathway	0.911	0.581
(9a) Cardiac adrenergic Signaling		
(9b) Fatty Acid oxidation	0.916	0.732
(10a) Human Embryonic Stem Cell Pluripotency		
(10b) Fatty Acid oxidation	0.926	0.615

**Table 3:** Differentially expressed miRNA-regulating pathway cross-talk. ex.BC indicates miRNA expression levels in BC and ex.NS indicates miRNA expression levels in NS.

Pairs Pathways	miRNA	logFC	ex.BC	ex.NS	Delta
(1a) Human Embryonic Stem Cell Pluripotency					
(1b) Putrescine Degradation III	*hsa-let-7c *	−1.507	12801.77	27353.99	21938.10
(2a) Intrinsic Prothrombin Activation Pathway					
(2b) Extrinsic Prothrombin Activation Pathway	*hsa-mir-210 *	3.273	2702.47	317.80	6433.72
(3a) Intrinsic Prothrombin Activation Pathway					
(3b) Extrinsic Prothrombin Activation Pathway	*hsa-mir-9-1 *	1.925	4679.87	1339.12	7806.52
(4a) Intrinsic Prothrombin Activation Pathway					
(4b) Extrinsic Prothrombin Activation Pathway	*hsa-mir-483 *	−1.786	22.80	67.85	28.86
(5a) Intrinsic Prothrombin Activation Pathway					
(5b) Extrinsic Prothrombin Activation Pathway	*hsa-mir-592 *	4.156	16.77	0.7	80.44
(6a) Ethanol Degradation IV					
(6b) Estrogen Receptor Signaling	*hsa-mir-103-2 *	1.134	48.07	19.65	32.24
(7a) Intrinsic Prothrombin Activation Pathway					
(7b) Extrinsic Prothrombin Activation Pathway	*hsa-mir-887 *	1.094	50.56	24.18	66.81
(8a) Acute Phase Response Signaling					
(8b) HIF1 Signaling	*hsa-mir-181b-2 *	1.432	23.47	8.02	22.14
(9a) Intrinsic Prothrombin Activation Pathway					
(9b) Extrinsic Prothrombin Activation Pathway	*hsa-mir-301b *	2.608	4.75	0.78	10.36
(10a) Intrinsic Prothrombin Activation Pathway					
(10b) Extrinsic Prothrombin Activation Pathway	*hsa-mir-147b *	2.312	3.99	0.701	7.61
(11a) Intrinsic Prothrombin Activation Pathway					
(11b) Extrinsic Prothrombin Activation Pathway	*hsa-mir-665 *	−1.447	2.28	5.56	3.88
(12a) Intrinsic Prothrombin Activation Pathway					
(12b) Extrinsic Prothrombin Activation Pathway	*hsa-mir-939 *	1.110	5.93	2.43	4.74

**Table 4:** Differentially expressed miRNA-regulating pathway cross-talk with mutual information: p.a ng indicates the number of genes for pathway a; mirna.p.a indicates the number of miRNA targets for pathway a; p.b ng indicates the number of genes for pathway b; mirna.p.b indicates the number of miRNA targets for pathway b.

Pairs Pathways	miRNA	p.a ng	mirna.p.a	p.b ng	mirna.p.b
(1a) Human Embryonic Stem Cell Pluripotency					
(1b) Putrescine Degradation III	*hsa-let-7c *	104	40	16	8
(2a) Intrinsic Prothrombin Activation Pathway					
(2b) Extrinsic Prothrombin Activation Pathway	*hsa-mir-9-1 *	19	9	12	4
(3a) Intrinsic Prothrombin Activation Pathway					
(3b) Extrinsic Prothrombin Activation Pathway	*hsa-mir-210 *	19	9	12	8
(4a) Intrinsic Prothrombin Activation Pathway					
(4b) Extrinsic Prothrombin Activation Pathway	*hsa-mir-592 *	19	5	12	4
(5a) Intrinsic Prothrombin Activation Pathway					
(5b) Extrinsic Prothrombin Activation Pathway	*hsa-mir-887 *	19	7	12	6
(6a) Ethanol Degradation IV					
(6b) Estrogen Receptor Signaling	*hsa-mir-103-2 *	17	4	112	15
(7a) Intrinsic Prothrombin Activation Pathway					
(7b) Extrinsic Prothrombin Activation Pathway	*hsa-mir-483 *	19	6	12	5
(8a) Acute Phase Response Signaling					
(8b) HIF1 Signaling	*hsa-mir-181b-2 *	146	26	94	19
(9a) Intrinsic Prothrombin Activation Pathway					
(9b) Extrinsic Prothrombin Activation Pathway	*hsa-mir-301b *	19	7	12	5
(10a) Intrinsic Prothrombin Activation Pathway					
(10b) Extrinsic Prothrombin Activation Pathway	*hsa-mir-147b *	19	5	12	5
(11a) Intrinsic Prothrombin Activation Pathway					
(11b) Extrinsic Prothrombin Activation Pathway	*hsa-mir-939 *	19	8	12	5
(12a) Intrinsic Prothrombin Activation Pathway					
(12b) Extrinsic Prothrombin Activation Pathway	*hsa-mir-665 *	19	7	12	4

**Table 5:** Principal target genes of miRNA-regulating pathway cross-talk in NS versus BC.

Pairs Pathways	miRNAs	Genes (a)	Genes (b)
(1a) Human Embryonic Stem Cell Pluripotency (1b) Putrescine Degradation III	*hsa-let-7c *	ACVR1 BMP4	ALDH1A1 ALDH1A21
		BMP5 BMP6	ALDH2 ALDH4A1
		FGF2 FZD7	ALDH9A1 IL4I1
		KLK3 LEFTY2	MAOA SMOX
		MRAS NTRK2	
		NTRK3 PDGFRA	
		PDGFRB PIK3CB	
		PIK3CD PIK3R6	
		S1PR1 SPHK1	
		TCF4 TCF7L1	
		TGFB1 TGFBR2	
		WNT11 WNT6	

(2a) Ethanol Degradation IV (2b) Estrogen Receptor Signaling	*hsa-mir-103-2 *	ACSS2 ALDH1A1	EP300 GTF2B
		ALDH3A1 ALDH9A1	GTF2H3 MED15
			MED17 MED6
			NCOR1 NCOR2
			PCK1 POLR2E
			TAF3 TAF7

(3a) Acute Phase Response Signaling (3b) HIF1 Signaling	*hsa-mir-181b-2 *	ELK1 HNRNPK	ARNT EGLN2
		IKBKB KRAS	EP300 EPO
		MAP2K7 MAPK1	KRAS MAPK1
		MAPK3 MRAS	MAPK3 MMP23B
		NRAS OSM	MRAS NRAS
		PIK3CB PIK3R2	PIK3CB PIK3R2
		PTPN11 SOCS4	SLC2A3 VHL
